# Tunable polymeric micelles for taxane and corticosteroid co-delivery

**DOI:** 10.1007/s13346-023-01465-x

**Published:** 2023-11-14

**Authors:** Armin Azadkhah Shalmani, Alec Wang, Zaheer Ahmed, Maryam Sheybanifard, Rahaf Mihyar, Eva Miriam Buhl, Michael Pohl, Wim E. Hennink, Fabian Kiessling, Josbert M. Metselaar, Yang Shi, Twan Lammers, Quim Peña

**Affiliations:** 1 Institute for Experimental Molecular Imaging, RWTH Aachen University Hospital, Forckenbeckstrasse 55, 52074 Aachen, Germany; 2 Electron Microscopy Facility, Institute of Pathology, RWTH University Hospital, Pauwelsstrasse 30, 52074 Aachen, Germany; 3 DWI – Leibniz-Institute for Interactive Materials, Forckenbeckstrasse 50, 52074 Aachen, Germany; 4 Department of Pharmaceutics, Utrecht Institute for Pharmaceutical Sciences, Faculty of Science, Utrecht University, 3508 TB Utrecht, The Netherlands

**Keywords:** Drug combination, Nanomedicine, Polymeric micelles, Co-loading, Taxane, Corticosteroid

## Abstract

Nanomedicine holds promise for potentiating drug combination therapies. Increasing (pre)clinical evidence is available exemplifying the value of co-formulating and co-delivering different drugs in modular nanocarriers. Taxanes like paclitaxel (PTX) are widely used anticancer agents, and commonly combined with corticosteroids like dexamethasone (DEX), which besides for suppressing inflammation and infusion reactions, are increasingly explored for modulating the tumor microenvironment towards enhanced nano-chemotherapy delivery and efficacy. We here set out to develop a size- and release rate-tunable polymeric micelle platform for co-delivery of taxanes and corticosteroids. We synthesized amphiphilic mPEG-*b*-p(HPMAm-Bz) block copolymers of various molecular weights and used them to prepare PTX and DEX single- and double-loaded micelles of different sizes. Both drugs could be efficiently co-encapsulated, and systematic comparison between single- and co-loaded formulations demonstrated comparable physicochemical properties, encapsulation efficiencies, and release profiles. Larger micelles showed slower drug release, and DEX release was always faster than PTX. The versatility of the platform was exemplified by co-encapsulating two additional taxane-corticosteroid combinations, demonstrating that drug hydrophobicity and molecular weight are key properties that strongly contribute to drug retention in micelles. Altogether, our work shows that mPEG-*b*-p(HPMAm-Bz) polymeric micelles serve as a tunable and versatile nanoparticle platform for controlled co-delivery of taxanes and corticosteroids, thereby paving the way for using these micelles as a modular carrier for multidrug nanomedicine.

**Graphical abstract:**

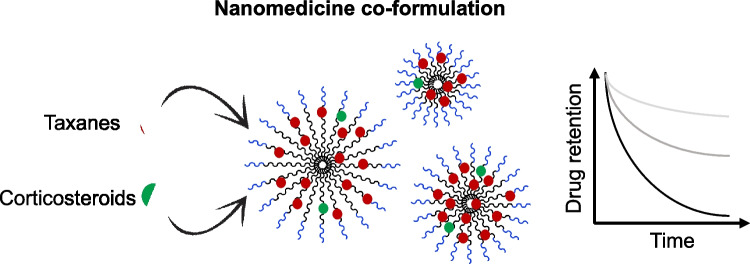

**Supplementary Information:**

The online version contains supplementary material available at 10.1007/s13346-023-01465-x.

## Introduction

Drug combination therapy is a mainstay in clinical oncology [1, 2]. Simultaneous employment of more than one drug can result in synergistic therapeutic efficacy and reduced toxicity [3]. Nanomedicines have demonstrated high value in potentiating such combination therapies by modulating biodistribution and pharmacokinetics of drugs [4]. More recently, employing nanomedicines for co-delivery of multiple drugs in a single platform has also shown to enhance combination therapy outcomes [5]. A prominent example of this is Vyxeos^®^, an FDA/EMA approved liposomal formulation co-encapsulating daunorubicin and cytarabine for the treatment of acute myeloid leukemia [6]. Besides enabling co-localization of (synergistic) drugs at the target site [7], co-loading more than one drug within a single nanocarrier can also facilitate nanomedicine pharmaceutical development and clinical translation as compared to the corresponding co-administered formulations (e.g., by simplifying manufacturing protocols and minimizing the number of toxicology assessments prior to entering clinical trials) [8].

Taxanes, particularly paclitaxel (PTX), are widely used chemotherapeutic drugs with approved clinical applications as part of combination therapies in different types of cancer [9–11]. Due to their high hydrophobicity and poor aqueous solubility, taxanes are formulated using surfactants or protein and polymeric nano-delivery systems to be administered to patients [12]. However, many cancers are notorious for having an abnormal and highly fibrotic tumor microenvironment (TME), constituted by a dense extracellular matrix (ECM) and a high interstitial fluid pressure (IFP) [13], which hinders effective access and delivery of taxane (nano)formulations to tumors [14]. To combat these hurdles, several pharmaceutical and pharmacological strategies have been proposed, including tuning the size of the nanocarriers [15, 16] or combining chemotherapy with TME-remodeling agents such as corticosteroids [17].

Corticosteroids, particularly dexamethasone (DEX), are routinely used as part of many cancer treatment regimens, either directly as anticancer agents or to attenuate the side effects of chemotherapeutics, including PTX [18, 19]. Other corticosteroids such as prednisolone (PRD) and its prodrug, prednisone, are commonly used in combination with the taxanes docetaxel (DTX) and cabazitaxel (CTX) as part of the treatment regimen for some types of prostate cancer [20, 21]. Recent studies have demonstrated that DEX is able to normalize blood vessels and to modulate the TME by reducing the content of ECM constituents (e.g., hyaluronic acid) and by lowering IFP, thus promoting nanomedicine penetration and improving anticancer efficacy [22]. More recently, co-loading taxanes with DEX in a single nanoparticulate delivery system was shown to significantly potentiate the overall therapy outcome [23, 24].

Given the high clinical relevance of combining taxanes and corticosteroids and the potential pharmacological, pharmaceutical, and translational benefits of co-formulating different drugs in a single nanocarrier, we see substantial value in developing modular nanomedicine platforms that are compatible with taxanes and corticosteroids and that are capable of controllably co-delivering them. Thus, we here aimed to design a size- and drug release rate-tunable nanocarrier platform for taxanes and corticosteroids combination therapy. By using such a drug combination, we furthermore set out to enhance our fundamental understanding of the impact of drug co-loading on key pharmaceutical properties of the resulting nanoformulations, such as size, polydispersity, and drug encapsulation efficiency and retention capabilities of the micelles [25]. To this end, we employed a micellar system based on methoxy poly(ethylene glycol)-*b*-(*N*-(2-benzoyloxypropyl) methacrylamide) (mPEG-*b*-p(HPMAm-Bz)) block copolymers. These amphiphilic block copolymers form micelles in an aqueous medium and solubilize drugs by encapsulating them in their cores through hydrophobic and π-π interactions. Importantly, they have already shown high potential as a delivery system for taxanes, with remarkable tumor suppression in different animal models [26, 27]. We initially prepared PTX and/or DEX single- and co-loaded micelles of different sizes and with varying drug feed amounts, and we systematically compared their physicochemical properties as well as drug encapsulation and retention. In order to validate the versatility of the nano-platform, we further sought out to extend by co-formulating other clinically approved taxanes (DTX and CTX) and corticosteroids (PRD and ciclesonide (CIC)). Pharmaceutical properties of these drug combinations were also evaluated. Finally, we assessed the influence of different structural and physicochemical properties of the drugs on their retention in the polymeric micelles under physiological conditions.

## Material and methods

### Materials

*N*,*N*-dimethylformamide (DMF), lithium chloride (LiCl), tetrahydrofuran (THF), acetonitrile (ACN), benzoyl chloride, *N*,*N*-dimethylpyridin-4-amine (DMAP), 4,4′-azobis(4-cyanopentanoic acid) (ABCPA), 4-(dimethylamino)pyridinium 4-toluenesulfonate (DPTS), *N*,*N*′-dicyclohexylcarbodiimide (DCC), diethyl ether (Et_2_O), deuterated dimethyl sulfoxide (DMSO-*d*_*6*_), trifluoroacetic acid (TFA), dichloromethane (DCM), poly(ethylene glycol) methyl ether (mPEG, M_n_ of 5000 Da), paclitaxel (PTX), docetaxel (DTX), cabazitaxel (CTX), prednisolone (PRD), dexamethasone (DEX), and ciclesonide (CIC) were purchased from commercial suppliers in synthesis grade purity and used as received. The solvents used for the syntheses were synthesis grade and dried on 4 Å molecular sieves, except when directly purchased in anhydrous form. HPMAm-Bz (monomer) and mPEG-ABCPA-mPEG (macroinitiator) were synthesized as previously reported [26].

### Synthesis of mPEG-*b*-p(HPMAm-Bz) block copolymers

Free radical polymerization was employed to synthesize mPEG-*b*-p(HPMAm-Bz) block copolymers using HPMAm-Bz as the monomer and mPEG-ABCPA-mPEG as the macroinitiator, following previously reported procedures with small modifications [26]. Briefly, the macroinitiator was synthesized through an esterification of mPEG and ABCPA, with DCC as a coupling reagent and DPTS as a catalyst [28]. After the reaction, the solution was cooled down to 0 °C and filtered off to remove the precipitated 1,3-dicyclohexyl urea, and the product was precipitated from the filtrate with cold Et_2_O (4 °C), collected through filtration, and dried under vacuum. Further purification was carried out by washing the solid with acetone, which removed the remaining reagent impurities. For polymerization, the monomer (300 mg/mL) and the macroinitiator were both dissolved in ACN. In order to obtain block copolymers of different molecular weights (by having different lengths of the hydrophobic block but identical hydrophilic (PEG) block), 3 different molar ratios of macroinitiator to monomer were used. Ratios of 1:100, 1:200, and 1:300 were used to synthesize copolymers of small, medium, and large sizes, respectively. The mixture solutions were degassed by purging with nitrogen for 20 min. The polymerization was carried out at 70 °C under nitrogen atmosphere for 18 h. Finally, the synthesized polymers were collected by precipitation in cold Et_2_O (×2) and dried under vacuum to obtain a white powder. Yields for small, medium, and large polymers were 86%, 68%, and 60%, respectively.

### Proton nuclear magnetic resonance (^1^H NMR) spectroscopy

Samples were dissolved in DMSO-*d*_*6*_, and ^1^H NMR spectra were obtained in Varian AV400 and AV600 instruments (Bruker Corporation). The resulting spectra were processed and analyzed using MestReNova 6.0. Degree of polymerization was determined by integrating the peak at 8 ppm, which corresponds to the aromatic protons of HPMAm-Bz, and the number average molecular weight (M_n_) based on ^1^H NMR was calculated as reported previously [26].

### Gel permeation chromatography (GPC)

GPC was performed to determine the number average molecular weight (*M*_n_), weight average molecular weight (*M*_w_), and dispersity (*Đ* = M_w_/M_n_) of the synthesized polymers. Samples were prepared by dissolving the polymers in DMF at a concentration of 2 mg/mL, and volumes of 45 µL were injected. A precolumn (PLgel 5 µm 50 × 7.5 mm, Agilent technologies) followed by two serial pLgel 5 µm MIXED-D columns (300 × 7.5 mm, Agilent technologies) were used to carry out the experiments. PEGs of different molecular weights and of narrow molecular weight distribution (Agilent Technologies) were used as calibration standards. DMF containing 10 mM LiCl was used as the eluent at a flow rate of 0.7 mL/min, and the columns were kept at a temperature of 55 °C. Detection was performed using a refractive index detector.

### High-performance liquid chromatography (HPLC)

Analytical reversed-phase HPLC was carried out using an Agilent HPLC system (1260 infinity II) equipped with a quaternary pump, a C18 column (4.6 × 150 mm, particle size 5 µm) and a UV-vis detector. A gradient elution method comprising ACN (containing 0.1% v/v TFA) and H_2_O (containing 0.1% v/v TFA) was used for PTX, DEX, DTX, PRD, CTX, and CIC. In the case of PTX and DEX, an injection volume of 25 µL (PTX) and 15 µL (DEX), a flow rate of 1 mL/min, and a detection wavelength of 242 nm were used (PTX retention time (R_t_), 3.6 min; DEX R_t_, 4.0 min). For DTX, PRD, CTX, and CIC (as well as PTX and DEX for the overlapped HPLC chromatograms in Fig. S10), an injection volume of 25 µL, a flow rate of 1 mL/min, and a detection wavelength of 230 nm were used (DTX R_t_, 11.8 min; PRD R_t_, 8.5 min; PTX R_t_, 12 min; DEX R_t_, 9.4 min; CTX R_t_, 13.1 min; and CIC R_t_, 16.4 min). The used solvent gradients for the different compounds are indicated in Tables S1–S3.

### Micelle preparation

Empty and drug-loaded polymeric micelles were prepared via a nanoprecipitation method [26]. To this end, 30 mg of small, medium, or large mPEG*-b-*p(HPMAm-Bz) block copolymers (and various drug amounts in case of the loaded micelles) were dissolved in 1 mL of THF. The solutions were dropwise added to 1 mL of Milli-Q water under vigorous stirring at 1000 rpm and kept on stirring for 1 min. The samples were then kept at RT for 24 h to allow evaporation of THF. Afterwards, the volume of the micellar dispersions was adjusted to 1 mL with Milli-Q water, and the dispersions were filtered through a 0.45-µm polyethersulfone (PES) filter.

To quantify encapsulation efficiency (EE) and loading capacity (LC), 50 µL of drug-loaded micelle dispersions was diluted in 450 µL of ACN. The amount of each drug was measured via HPLC using the abovementioned procedures. EE and LC were calculated using Eqs. 1 and 2, respectively:

1 $$\mathrm{ EE }\,\left(\mathrm{\%}\right)= \frac{\text{weight of the drug loaded into the micelles}}{\text{feed weight of the drug}}\times 100\mathrm{ \%}$$

2 $$\mathrm{ LC }\,\left(\mathrm{\%}\right)= \frac{\text{weight of the drug loaded into the micelles}}{\text{weight of the loaded drugs and the polymer}}\times 100\mathrm{ \%}$$

### Dynamic light scattering (DLS)

Measurement of the hydrodynamic diameter (size, Z-average), polydispersity index (PDI), and zeta potential of the formulations was performed using a DLS instrument (Nano-S, Malvern Panalytical PLC). For determining the size and PDI, micellar dispersions were diluted in Milli-Q water to a polymer concentration of 300-1000 µg/mL and transferred into disposable polystyrene cuvettes before measurement. The samples were measured at a fixed scattering angle of 173° and a temperature of 25 °C while the attenuator was set to automatic. For zeta potential measurements, samples were diluted to a polymer concentration of between 5 and 15 mg/mL.

### Transmission electron microscopy (TEM)

For TEM analysis, micellar dispersions (30 mg/mL) were diluted 200 times with Milli-Q water. The samples were left to adsorb onto 200-mesh glow discharged formvar-carbon-coated nickel grids (Maxtaform) for 10 min, followed by 3 rounds of washing with Milli-Q water. Negative staining of the samples was carried out with 0.5% uranyl acetate (Science Services GmbH). Samples were imaged using a LEO 906 (Carl Zeiss) microscope at an acceleration voltage of 60 kV.

### Critical micelle concentration (CMC)

Pyrene, as fluorescent probe, was used to determine the CMC of the different mPEG-*b*-p(HPMAm-Bz) block copolymers [29, 30]. Serial dilutions of micelles (from 100 to 0.4 µg/mL) were prepared in water. Next, 6 µL of pyrene in acetone (0.18 mM) was added to 1.5 mL of the micellar dispersions, and the mixtures were incubated at RT in the darkness for 20 h. Thereafter, fluorescence excitation spectra of pyrene were recorded using a spectrofluorometer (Tecan infinite m200 pro) at a 90° angle. The excitation spectra (300 to 360 nm with emission wavelength of 390 nm) were recorded while excitation and emission band slits were set to 4 and 2 nm, respectively. The ratio of excitation intensities at 338 nm to 333 nm was plotted against the concentration of the polymer to determine the CMC.

### Drug release

Release profile of the different loaded drugs from the micelles was assessed under sink conditions. A solution of 45 mg/mL bovine serum albumin (BSA) in phosphate-buffered saline (PBS, composition: 137.9 mM sodium chloride, 1.47 mM potassium phosphate monobasic, 2.67 mM potassium chloride, 8.09 mM sodium phosphate dibasic) at pH 7.4 as the medium and Float-A-Lyzer dialysis devices (MWCO of 300 kDa) were employed for this purpose as reported previously [31]. Briefly, the micellar dispersions were transferred into the dialysis devices (1 mL), subsequently submerged in the medium and kept at 37 °C under shaking agitation. At each time point, 50 µL of samples was taken from the dialysis device. The taken volumes from the dialysis devices at every time point were compensated with 50 µL of the medium (PBS pH 7.4 containing 45 mg/mL of BSA). The samples were diluted 10 times in ACN and centrifuged at 5000 g for 10 min to remove the precipitated BSA. Finally, drug content in the supernatant was determined via HPLC, following the previously described method for each drug.

### Micelle stability

To evaluate micelle stability and drug retention at two different pH conditions (7.4 and 6), the co-loaded formulations (diluted 6 times to a final volume of 1 mL) were transferred into Float-A-Lyzer dialysis devices (MWCO of 300 kDa). The devices were subsequently submerged into 40 mL of PBS solution. For pH 7.4, a PBS solution with the following composition was used: 137.9 mM sodium chloride, 1.47 mM potassium phosphate monobasic, 2.67 mM potassium chloride, and 8.09 mM sodium phosphate dibasic. For pH 6, HCl was used to adjust the pH of the PBS solution. Micelles at both pH conditions were incubated for 7 days at room temperature (RT). Upon each time point, 100 µL of the inner medium (samples inside the dialysis devices) was withdrawn and compensated with the same volume of the PBS solution at the corresponding pH. The withdrawn samples were used to measure both micelle size and PDI changes as well as drug retention. For drug content quantification, the samples were diluted 10 times in ACN to disrupt the micelles before HPLC analysis. HPLC and DLS measurements were performed as described in the corresponding sections.

### Statistical analysis

Statistical analyses were performed using GraphPad Prism software version 9.0. Associations between different drug properties and its retention in the micelles were assessed using simple and multiple linear regression.

## Results and discussion

**Fig. 1:**
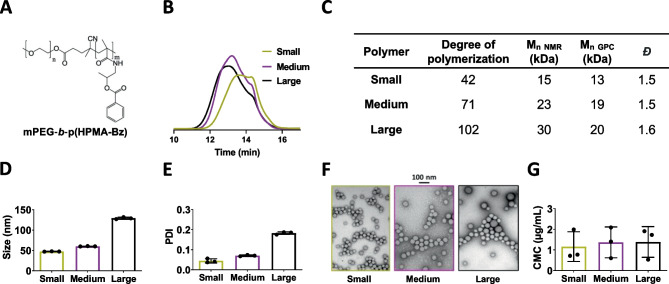
Preparation of polymeric micelles of different sizes. **A** Chemical structure of mPEG‐*b*‐p(HPMAm-Bz) block copolymers. **B** GPC chromatograms of small, medium, and large polymers. **C** Characterization of small, medium, and large polymers by ^1^H NMR and GPC, where degree of polymerization refers to the number of HPMAm-Bz units in the hydrophobic block. **D**, **E** Size (**D**) and polydispersity index (PDI) (**E**) of micelles prepared from small, medium, and large mPEG‐*b*‐p(HPMAm-Bz) copolymers. **F** TEM images of micelles prepared from small, medium, and large mPEG‐*b*‐p(HPMAm-Bz) copolymers. **G** Critical micelle concentration (CMC) of the three polymers. To prepare 1 mL of micellar dispersion, 30 mg of polymer were used. Data are presented as mean ± SD (*N* = 3)

Tuning micelle size was achieved by tailoring the molecular weight of their constituent polymers. To this end, we synthesized mPEG-*b*-p(HPMAm-Bz) block copolymers with a fixed molecular weight of the hydrophilic PEG block (5 kDa) and a varying molecular weight of the hydrophobic HPMAm-Bz block. The hydrophobic blocks of the resulting polymers had degrees of polymerization of 42, 71, and 102 (here referred to as small, medium, and large, respectively) based on NMR (Figs. 1A, C and S1), which corresponded with an overall number average molecular weight of 15.4, 22.5, and 30.2 kDa. The three polymers demonstrated similar dispersity based on GPC analysis (*Đ* = 1.5–1.6, Fig. 1B and C). The small, medium, and large polymers were separately used to prepare non-drug loaded micelles of different sizes, resulting in self-assemblies of 47, 60, and 130 nm size, respectively (Fig. 1D). All the micelles had a rather narrow size distribution with PDI around or below 0.2. Our DLS results corroborated a unimodal size distribution for all of them (Fig. S2). TEM images confirmed that the micelles, regardless of the size, had similarly spherical morphology and a narrow size distribution (Fig. 1F).

Colloidal stability of the micelles was assessed by determining the CMC of their polymers. Low CMC values are crucial to ensure preservation of the self-assembled structure and retention of drugs upon dilution in the bloodstream after formulation administration [32]. Micelles prepared from the three different polymers demonstrated high stability with CMC values between 1 and 2 µg/mL (Fig. 1G), which were similar to previous observations [26, 33].

**Fig. 2:**
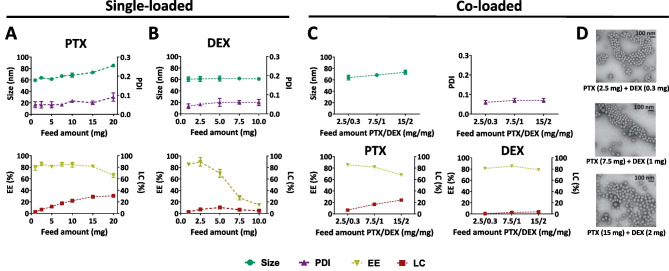
Preparation and characterization of paclitaxel and dexamethasone single- and co-loaded medium-sized micelles. **A**, **B** Size, polydispersity index (PDI), encapsulation efficiency (EE), and loading capacity (LC) of paclitaxel (PTX) single-loaded (**A**) and dexamethasone (DEX) single-loaded (**B**) micelles at different drug feed amounts. **C** Size, polydispersity index (PDI), drug encapsulation efficiency (EE), and loading capacity (LC) of PTX and DEX co-loaded micelles with different feed amounts. **D** TEM images of PTX and DEX co-loaded micelles at different drug feed amounts. To prepare 1 mL of micellar dispersion, 30 mg of polymers were used in all cases. Data are presented as mean ± SD (*N* = 3)

Subsequently, we prepared PTX and DEX single- and co-loaded micelles of different sizes (small, medium, and large) and evaluated the effect of co-encapsulation on key pharmaceutical properties such as size, size distribution, and drug encapsulation. To do so, we comparatively assessed the impact of increasing drug feed amounts and drug type in both single- and co-loaded setups, initially using medium-sized micelles (Fig. 2A).

For single-loaded PTX micelles, a wide range of 1 to 20 mg of PTX feed amount was examined. The micelles efficiently solubilized PTX with encapsulation efficiencies (EE) of about 80% for up to 15 mg of PTX and a maximum loading capacity (LC) of 30%. For higher PTX feed amounts (20 mg), the EE dropped to about 65%. Increasing PTX amounts was also accompanied by a gradual increase in micelle size from 60 to 80 nm, while the narrow size distribution of the micelles was maintained with PDI values < 0.1. For single loading DEX into the medium-sized micelles (Fig. 2B), a feed amount range of 0.3 to 10 mg was used, as the clinically used doses for DEX are remarkably lower for DEX compared to PTX [34–36]. Contrary to the single-loaded PTX micelles, increasing DEX feed amount had no effect on the size and size distribution of the formulations (~ 60 nm and PDI < 0.1). While high EE values of 80–90% were achieved at low DEX feed amounts (0.3–2.5 mg), the EE values drastically decreased to about 20% when feed amounts above 5 mg were used, due to significant DEX precipitation. The maximum LC of DEX-loaded micelles was approximately 10%. Single-loaded micelles with different drug feed amounts were also prepared from small and large polymers (Figs. S3 and S4). The different loaded micelles showed similar sizes and PDI as their empty counterparts. Except for 15 mg PTX feed in small polymers, all the formulations had EE values of 80% or higher.

For the PTX and DEX co-loaded micelles using medium-sized polymers, we employed a fixed PTX-to-DEX feed amount ratio of around 7 to 1 (w/w). This ratio was selected considering that the commercially available PTX formulations have drug concentrations between 5 and 6 mg/mL and that the clinical dose of DEX is lower than that of PTX [34–37]. We used three different PTX/DEX feed amounts of 2.5/0.3, 7.5/1, and 15/2 (mg/mg) (Fig. 2C, D). The size of the micelles slightly increased from 65 to 75 nm with increasing total drug feed amount, while PDI remained below 0.1 in all the cases (Fig. 2C). The increase in size is likely caused by the higher feed amount of PTX. Furthermore, the zeta potential of the PTX-DEX co-loaded micelles (with 7.5/1 (mg/mg) PTX/DEX feed amounts) was measured and compared to that of the empty formulation. In both cases, the micelles were found to be slightly negatively charged, with zeta potential values in the range of − 1 to − 2 mV (Fig. S5). Nanoparticles with neutral or slightly negative surface charge have been reported to circulate longer in the bloodstream and show improved tumor accumulation as compared to highly (positively) charged particles [38].

Regarding drug encapsulation, DEX was efficiently loaded (EE of 80%) in all three co-loaded formulations, whereas EE values for PTX only slightly decreased at high feed amounts (15 mg) with respect to the corresponding single-loaded micelles (from about 80 to 70%). LC for PTX and DEX in the assessed range had maximum values of around 25% and 5%, respectively. TEM images of all three co-loaded formulations confirmed homogeneous spherical morphology (Fig. 2D). Overall, PTX-DEX co-loaded micelles demonstrated analogous physiochemical properties to the PTX single-loaded ones, probably due to the significantly higher feed amount of PTX as compared to DEX. Furthermore, EE values for both drugs in the co-loaded formulations were comparable to the single-loaded counterparts.

**Fig. 3:**
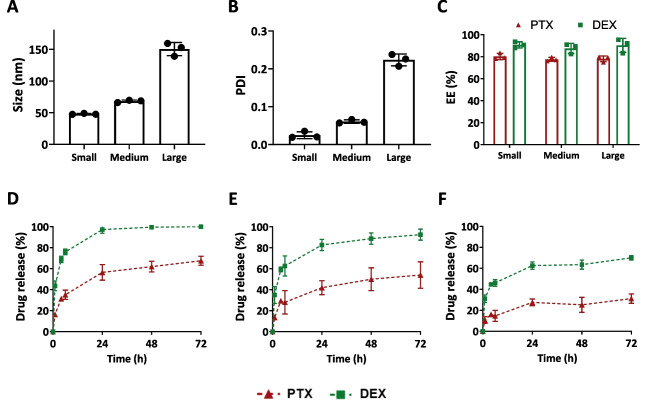
Comparison of paclitaxel and dexamethasone co-loaded micelles of different sizes. **A**, **B** Size (**A**) and polydispersity (PDI) (**B**) of paclitaxel (PTX) and dexamethasone (DEX) co-loaded small-, medium-, and large-sized micelles. **C** PTX and DEX encapsulation efficiency (EE). **D–F** PTX and DEX release profile of micelles prepared from small (**D**), medium (**E**), and large (**F**) polymers in simulated physiological conditions (sink conditions, PBS pH 7.4 containing 45 mg/mL BSA). For all the formulations, 30 mg of polymers and PTX/DEX feed amount of 7.5/1 (mg/mg) were used to prepare 1 mL of micellar dispersion. Data are presented as mean ± SD (*N* = 3)

To assess the influence of the polymer molecular weight on the pharmaceutical properties of the corresponding co-loaded formulations, micelles of different sizes with a PTX/DEX feed amount of 7.5/1 (mg/mg) were prepared. As observed for empty and single-loaded micelles, the size of the co-loaded formulations was mostly driven by the molecular weight of their constituting block copolymers, with values of about 50, 70, and 150 nm for small, medium, and large polymers, respectively (Fig. 3A). Co-loaded micelle sizes were similar to PTX single-loaded formulations and slightly larger than the empty ones, likely due to the solubilization of the drugs in the hydrophobic core of the micelles. PDI values followed a similar trend in both non-loaded and single-loaded micelles, indicating that all the formulations had narrow size distributions (Fig. 3B). Both PTX and DEX were efficiently loaded in the three different micelles, with EE of about 80% for PTX and 90% for DEX (Fig. 3C).

Drug release in physiological conditions was studied by placing different formulations in a dialysis setup under sink conditions and using 45 mg/mL BSA in PBS (pH 7.4) solution as medium over the course of 72 h (Fig. 3D–F). A similar experimental setup was recently reported to be highly representative of in vivo drug retention in mice [39]. Drug retention capabilities of the micelles increased as the molecular weight of hydrophobic block became larger (i.e., large > medium > small polymers). After 24 h, about 55, 45, and 30% of PTX was released from small, medium, and large micelles, respectively, and a similar trend was observed for DEX (from 95% for small, to 65% for large micelles), which is in line with previous observations [31, 33]. Interestingly, DEX was released about two times faster than PTX regardless of the micelle size, which can be caused by a weaker interaction of DEX with the core of the micelles. This kinetically controlled release pattern can contribute to achieving sequential pharmacological effects, which may be favorable in multiple therapeutic setups. The released DEX could prime the TME to promote deeper micelle penetration, thereby enabling more effective PTX delivery to and action at the pathological site. Such sequential effects, achieved through different release rates for two drugs co-loaded in a single carrier, have been previously shown to be beneficial for improving antitumor response and therapeutic index [40].

To understand whether co-loading two drugs influences the release profile of the individual compounds, PTX and DEX single-loaded micelles of the three different sizes were also assessed for drug retention in PBS medium with BSA (Figs. S6–S8). Both PTX and DEX in all single-loaded formulations demonstrated similar release behavior in comparison to their co-loaded counterparts. Furthermore, the feed amount of the encapsulating drug did not play a remarkable role in the drug release profile. All in all, a modular nanoplatform for kinetically controlled co-delivery of PTX and DEX was successfully developed. Additionally, the data obtained for the co-loaded versus single-loaded micelles suggest that co-encapsulation of both drugs does not influence the retention of the individual PTX and DEX molecules in the hydrophobic core of the micelles and thereby does not impact the pharmaceutical properties as compared to the single-loaded formulations.

**Fig. 4:**
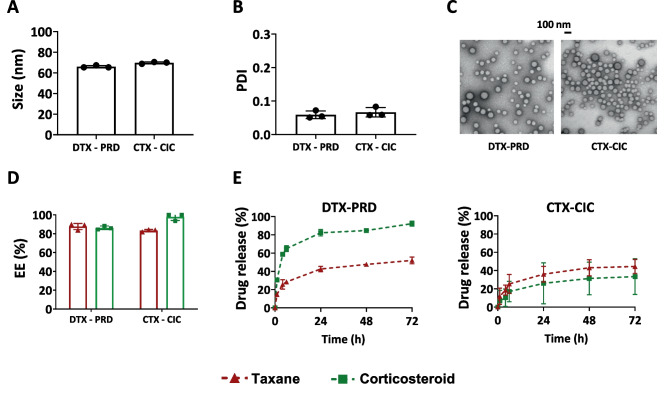
Preparation and characterization of two other taxane-corticosteroid co-loaded micelles. Docetaxel with prednisolone (DTX-PRD) and cabazitaxel with ciclesonide (CTX-CIC) co-loaded micelles were prepared using mPEG-*b*-p(HPMAm-Bz) of medium molecular weight. **A**, **B** Size (**A**) and polydispersity index (PDI) (**B**) of DTX-PRD and CTX-CIC co-loaded micelles. **C** TEM images of DTX-PRD and CTX-CIC co-loaded micellar dispersions. **D** Drug encapsulation efficiency (EE) for DTX-PRD and CTX-CIC micelles. **E** Drug release profile of DTX-PRD and CTX-CIC micelles. A total of 30 mg of polymers and a taxane/corticosteroid feed amount of 7.5/1 (mg/mg) were used to prepare 1 mL of micellar dispersions. Data are presented as mean ± SD (*N* = 3)

As the next step, we aimed to assess the versatility of the co-loaded platform beyond PTX and DEX, to two other clinically approved taxanes (DTX and CTX) and corticosteroids (PRD and CIC). To this end, we chose the least and the most hydrophobic taxane-corticosteroid combinations based on log P (Table 1), i.e., DTX-PRD and CTX-CIC, respectively. Medium-sized polymers with a feed of 7.5 mg for taxanes and 1 mg for corticosteroids were used to prepare micelles. The resulting micelles showed analogous size (around 70 nm) and PDI (below 0.1), as well as similarly homogenous spherical morphology among both drug combinations (Fig. 4A–C), as well as to the PTX-DEX micelles. The zeta potentials of DTX-PRD and CTX-CIC co-loaded micelles were also measured. Nanoparticles from both formulations showed slightly negative surface charges, with values in the range of − 1 to − 2 mV, similar to empty and PTX-DEX co-loaded micelles (Fig. S5). The loading of different drugs in the micelles, thus, did not influence the surface charge of the formed nanoparticles. Moreover, both DTX-PRD and CTX-CIC were also efficiently co-loaded in the micelles, with EE higher than 80% in all the cases (Fig. 4D). Drug release profiles of the micelles were evaluated under sink conditions in a medium containing BSA (Fig. 4E). As for PTX-DEX co-loaded micelles, the release rate for PRD (corticosteroid) was substantially faster than that of DTX (taxane), endowing the formulation with a pharmacologically favorable release feature that can result in advantageous sequential therapeutic effects. Conversely, CTX-CIC co-loaded micelles displayed a different release pattern between the two drug classes as compared to the previous co-encapsulated formulations; while CTX showed similar release kinetics to the two other taxanes, CIC release from the micelles was remarkably slower than the two other corticosteroids (DEX and PRD) and similar to CTX. This behavior may be ascribed to the high hydrophobicity of CIC (log P value of 4).

**Table 1:**
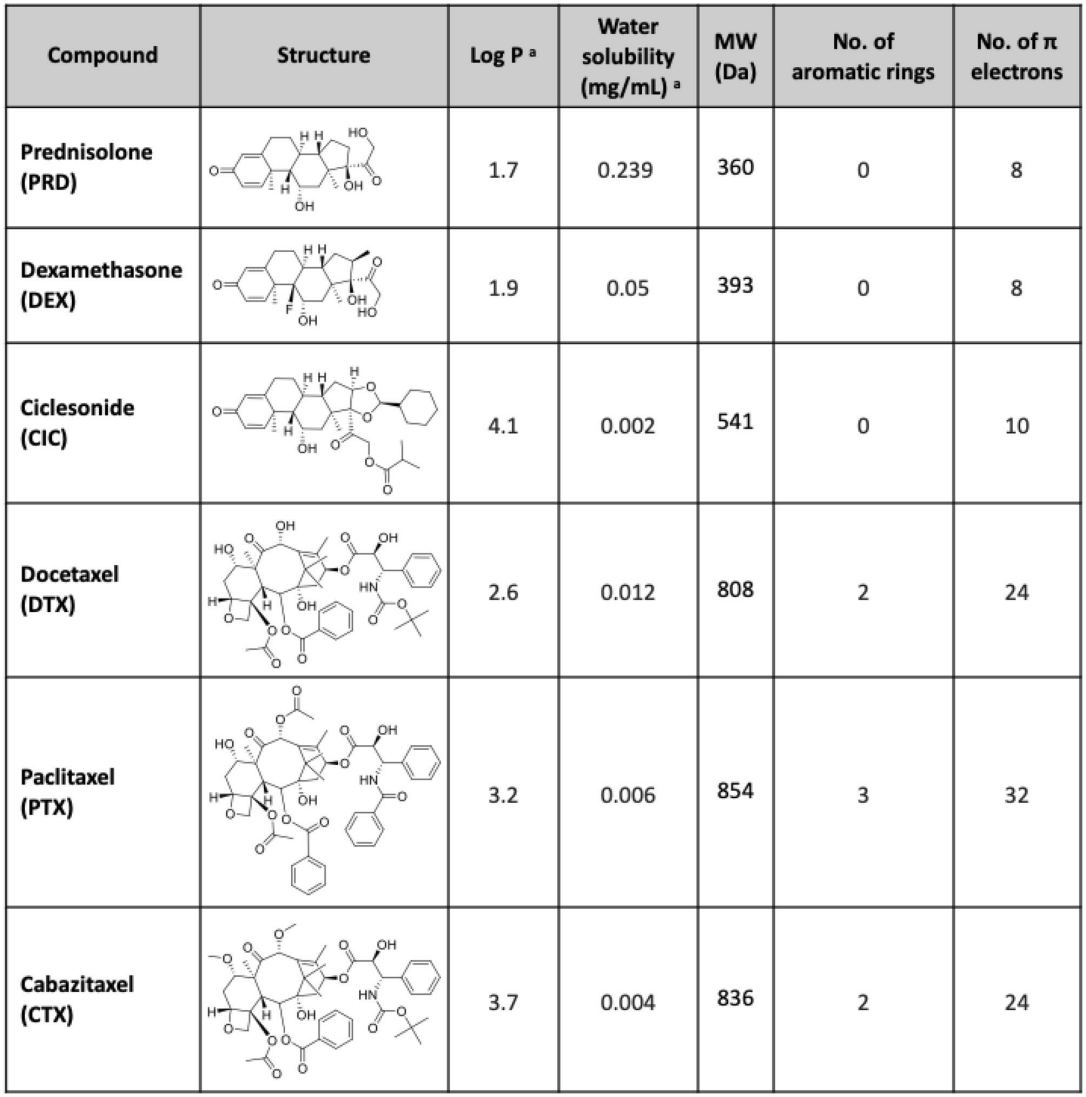
Structural and physicochemical properties of the three corticosteroids (prednisolone (PRD), dexamethasone (DEX), and ciclesonide (CIC)) and taxanes (docetaxel (DTX), paclitaxel (PTX), and cabazitaxel (CTX)) that were co-loaded in mPEG-*b*-p(HPMAm-Bz) micelles

^a^Values for log P and water solubility were obtained from DrugBank (based on ALOGPS, except for prednisolone, which is based on Chemaxon source) [41]

To explore whether different biologically relevant pH conditions influence polymeric micelle stability and drug retention, the three co-loaded formulations (prepared using medium polymers with 7.5 mg of taxane and 1 mg of corticosteroid as feed amounts) were incubated in PBS-containing media at pH 7.4 (representing bloodstream) and 6 (representing the TME and endosomes) for 7 days (Fig. S9). Our results showed that all co-loaded micelles were stable in terms of size and PDI over time in both pH conditions, without any signs of aggregation. Drug retention over time followed a similar trend to that observed previously in the sink condition release study, without a clear effect of the pH on the release behavior. For both pH 7.4 and 6, taxanes were better retained in micelles than corticosteroids, except for the CIC-CTX pair.

Taken together, we show that the mPEG-*b*-p(HPMAm-Bz)-based polymeric micelles comprise a versatile and tunable nanoparticle platform for corticosteroids and taxanes co-delivery. Therefore, understanding which drug properties affect the retention in the delivery system can assist in optimizing nanomedicine design. A recent study by Varela-Moreira et al*.* [39] aimed to shed light on this matter by evaluating the micellar retention of 4 different compounds of various drug categories. Their results suggest that while the log P is important for drug encapsulation into and retention in the micelles, the number of aromatic rings of the drug is more strongly associated with its retention. Given the high retention observed for CIC in the present work, we aimed to deepen into this question using the findings from the three taxanes and three corticosteroids.

**Fig. 5:**
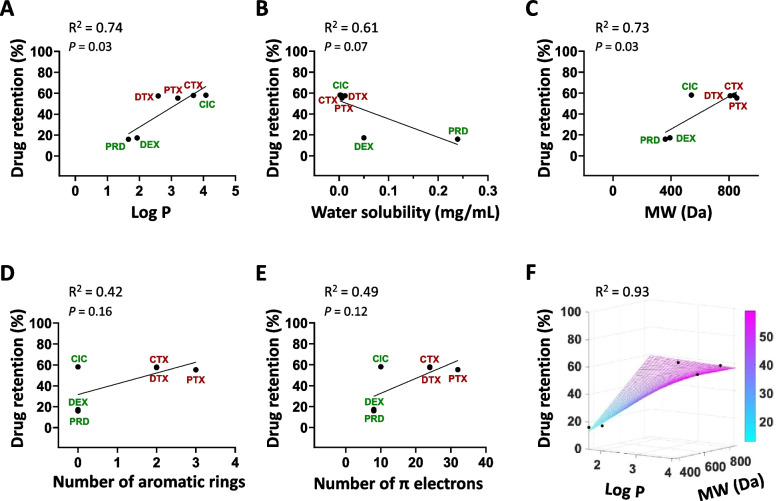
Associations between taxane and corticosteroid properties and their retention in micelles. **A–E** Drug retention in mPEG-*b*-p(HPMAm-Bz) polymeric micelles after 24 h as a function of log P (**A**), water solubility (**B**), molecular weight (MW) (**C**), number of aromatic rings (**D**), and number of π electrons (**E**). **F** Drug retention after 24 h as a function of both log *P* and MW (colored bar indicates drug retention)

Considering that neither the feed amount of the loaded drugs nor their state of being single- or co-loaded into the micelles influenced their retention, we analyzed the obtained drug release data in order to evaluate the association between different structural and physicochemical properties of the drug and its retention in the micelles. The properties of the used taxanes and corticosteroids are provided in Table 1. HPLC chromatograms of all the 6 compounds using a non-polar C18 column (Fig. S10) confirmed the highly hydrophobic nature of CIC compared to the other compounds. To understand which drug property contributes best to its micellar retention, we plotted the percentage of drug retained in the micelles after 24 h as a function of different drug characteristics (log P, water solubility, molecular weight, number of aromatic rings, and number of π electrons) using single linear regression (Fig. 5A–E). Among the properties evaluated, log P and MW showed the strongest associations with drug retention in the micelles (*R*^2^ > 0.7), followed by water solubility (*R*^2^ > 0.6). Interestingly, our findings point to a weaker effect of the number of aromatic rings on the drug retention (*R*^2^ = 0.42) for taxanes and corticosteroids compared to previous observations for another group of drugs [39]. In our case, this result is greatly influenced by CIC, which does not contain aromatic rings but is efficiently retained in the micelles. Further analysis using drug retention values at a shorter time (after 6 h) also confirmed similar trends, with drug retention being best associated with log P values (*R*^2^ > 0.8, Fig. S11).

Based on these data, we subsequently aimed to assess the combined effect of multiple drug properties on its retention in the micelles using multiple linear regression. The resulting analysis showed high multi-collinearity among the different properties assessed, which made the eventual modeling unreliable. To avert the problem, we only focused on log P and molecular weight, mainly as they are non-related properties and they both individually showed the best association with drug retention at 24 h. The effect of the two properties together on drug retention was analyzed using multiple linear regression (Fig. 5F), and the results showed a very high coefficient of determination (*R*^2^ > 0.9) and a low variance inflation factor (VIF) value (1.49). While having a high number of aromatic rings contributes to retention of the drugs in the mPEG-*b*-p(HMPAm-Bz) micelles, our findings demonstrate that it is certainly not a prerequisite. In addition, while it is important to take into account that statistically robust and predictive models cannot be obtained using our relatively small sample size (with only 6 drugs), our data do point towards a major contribution of molecular weight and particularly hydrophobicity (mainly log P, but also water solubility) to better drug retention; more prominently than previously assumed [39]. Collectively, it seems evident that drug retention in the micelles is not determined by only one individual feature. A combination of different structural and physiochemical properties modulates the interaction of the drug with the polymeric micelles, and the extent of each factor’s individual contribution can differ between various drug classes.

## Conclusion

Taken together, π electron-stabilized mPEG-*b*-p(HPMAm-Bz) polymeric micelles hold potential as a versatile and tunable co-delivery platform for taxanes and corticosteroids. Co-encapsulation of taxanes and corticosteroids does not impact the physicochemical properties, drug encapsulation efficiency, and drug retention capability of the micelles compared to single-loaded counterparts. By modulating the hydrophobic block length of the polymers, nanoparticle size and drug release rates could be controlled. The co-encapsulated taxane-corticosteroid pairs with the highest clinical relevance (i.e., PTX-DEX and DTX-PRD) exhibited differential release kinetics for the two drug classes. The faster release kinetics of corticosteroids in comparison to taxanes is a favorable formulation feature, which may help achieve pharmacologically advantageous sequential therapy effects for taxane-corticosteroid combinations. Finally, evaluating the impact of different drug properties on its micellar retention uncovered that both hydrophobicity and molecular weight most strongly contribute to taxanes and corticosteroids retention in the micelles. Our work establishes mPEG-*b*-p(HPMAm-Bz) micelles as a suitable nano-platform for drug combination therapy and sets the stage for co-formulation and co-delivery of other clinically relevant drugs. Overall, promoting more systematic evaluation and understanding of nano-pharmaceutical features for drug co-formulation can assist in unlocking the full potential of nanomedicine in the context of anticancer combination therapies, as well as contribute to the development of the next-generation multidrug nanomedicines by improving drug efficacy, streamlining pharmaceutical development processes, and fostering clinical translation.

## Supplementary Information

Below is the link to the electronic supplementary material.

Supplementary file1 (DOCX 736 KB)

## Data Availability

Data are available upon reasonable request.
