# Supplementary material for: Tunable polymeric micelles for taxane and corticosteroid co-delivery
Source: Drug Deliv Transl Res. 2023 Nov 14;14(10):2642–54. doi: 10.1007/s13346-023-01465-x (PMC11385023; doi:10.1007/s13346-023-01465-x)
Supplement: Supplementary file 1 — Supplementary file1 (DOCX 736 KB) [file 13346_2023_1465_MOESM1_ESM.docx]

**- Supporting information -**

**Tunable polymeric micelles for taxane and corticosteroid co-delivery**

Armin Azadkhah Shalmani ^1^, Alec Wang ^1^, Zaheer Ahmed ^1^, Maryam Sheybanifard ^1^,

Rahaf Mihyar ^1^, Eva Miriam Buhl ^2^, Michael Pohl ^3^, Wim E. Hennink ^4^, Fabian Kiessling ^1^,

Josbert M. Metselaar ^1^, Yang Shi ^1^, Twan Lammers ^1,*^, Quim Peña ^1,*^

*^1^ Institute for Experimental Molecular Imaging, RWTH Aachen University Hospital, Forckenbeckstrasse 55, 52074 Aachen, Germany*

*^2^ Electron Microscopy Facility, Institute of Pathology, RWTH University Hospital, Pauwelsstrasse 30, 52074 Aachen, Germany*

*^3^ DWI – Leibniz-Institute for Interactive Materials, Forckenbeckstrasse 50, 52074 Aachen, Germany*

*^4^ Department of Pharmaceutics, Utrecht Institute for Pharmaceutical Sciences, Faculty of Science, Utrecht University, 3508 TB Utrecht, The Netherlands*

^*^ *Corresponding authors: Twan Lammers (*[*tlammers@ukaachen.de*](mailto:tlammers@ukaachen.de)*), Quim Peña (*[*jpena@ukaachen.de*](mailto:jpena@ukaachen.de)*)*

1. **Supplementary tables:**

**Table S1. HPLC solvent gradient for paclitaxel (PTX).**

| **PTX** | | |
| --- | --- | --- |
| **Time (min)** | **ACN % (v/v)** | **Water % (v/v)** |
| 0 | 56 | 44 |
| 3 | 66 | 34 |
| 3.01 | 100 | 0 |
| 6 | 100 | 0 |
| 6.01 | 56 | 44 |
| 9 | 56 | 44 |

**Table S2. HPLC solvent gradient for dexamethasone (DEX).**

| **DEX** | | |
| --- | --- | --- |
| **Time (min)** | **ACN % (v/v)** | **Water % (v/v)** |
| 0 | 38 | 62 |
| 3 | 48 | 52 |
| 3.01 | 100 | 0 |
| 6 | 100 | 0 |
| 6.01 | 38 | 62 |
| 9 | 38 | 62 |

**Table S3. HPLC solvent gradients for docetaxel (DTX), prednisolone (PRD), cabazitaxel (CTX), and ciclesonide (CIC).**

| **DTX, PRD, CTX, CIC, (and PTX and DEX in the overlapped chromatogram)** | | |
| --- | --- | --- |
| **Time (min)** | **ACN % (v/v)** | **Water % (v/v)** |
| 0 | 0 | 100 |
| 15 | 100 | 0 |
| 18 | 100 | 0 |
| 21 | 0 | 100 |

*Note:* all HPLC solvents contained 0.1% of trifluoroacetic acid (TFA).

1. **Supplementary figures:**

**Figure S1. ^1^H NMR (DMSO-*d_6_*, 400 MHz) spectra of polymers of different sizes.** The peaks at 2.1, 2.5, and 3.3 ppm correspond to residual ACN, DMSO, and water, respectively. M_n NMR_ of the copolymers were estimated based on the integration of the peak at 8 ppm.

**Figure S2. Size distribution of micelles prepared from small, medium, and large mPEG-*b*-p(HPMAm-Bz)** **polymers measured by DLS.**

**
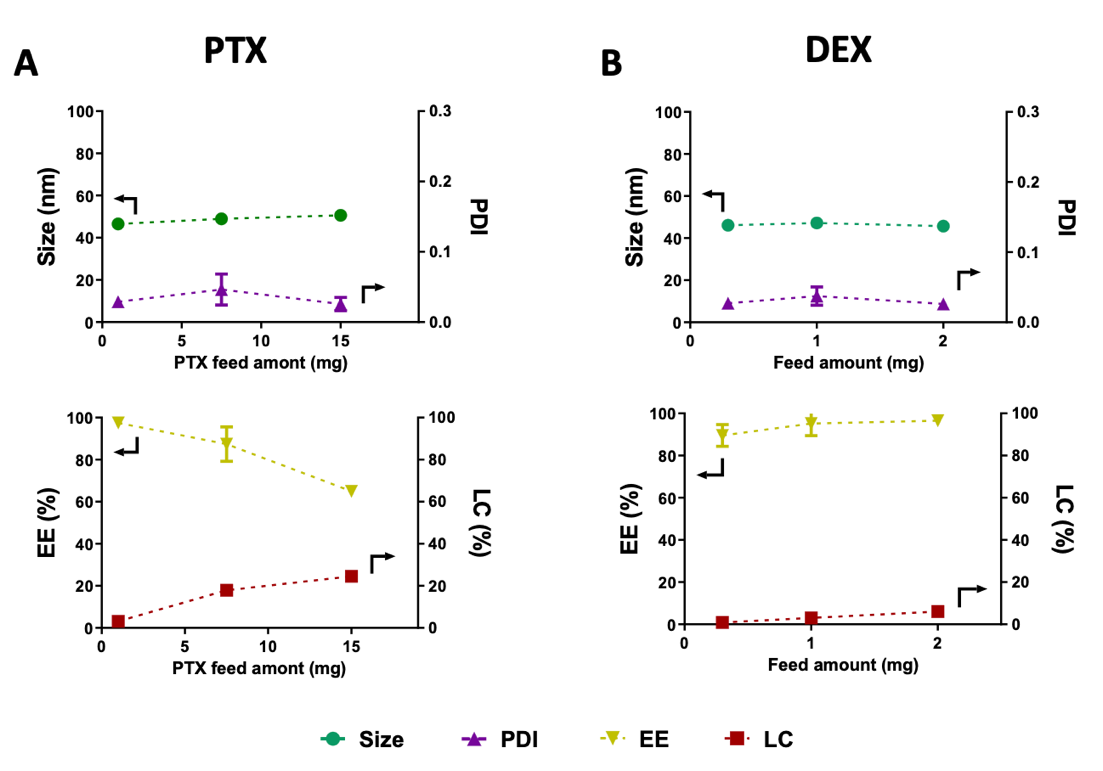
**

**Figure S3. Paclitaxel or dexamethasone single-loaded micelles prepared from small polymers.** Size, PDI, encapsulation efficiency (EE) and loading capacity (LC) of paclitaxel (PTX)-loaded (**A**) and dexamethasone (DEX)-loaded (**B**) micelles with different drug feed amounts. For all the formulations, 30 mg of polymer were used to prepare 1 mL of micellar dispersion.

**
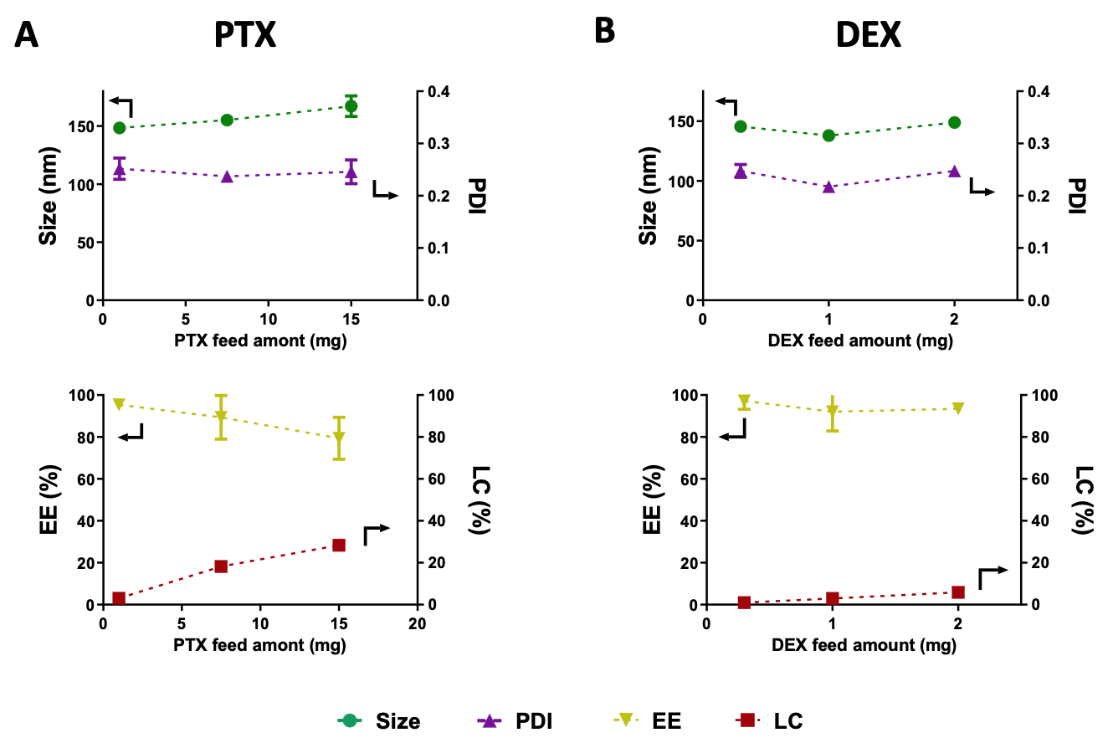
**

**Figure S4. Paclitaxel or dexamethasone single-loaded micelles prepared from large polymers.** Size, PDI, encapsulation efficiency (EE) and loading capacity (LC) of paclitaxel (PTX)-loaded (**A**) and dexamethasone (DEX)-loaded (**B**) micelles with different drug feed amounts. For all the formulations, 30 mg of polymer were used to prepare 1 mL of micellar dispersion.

**Figure S5. Zeta potential of empty and co-loaded micelles prepared from medium polymers.**

**
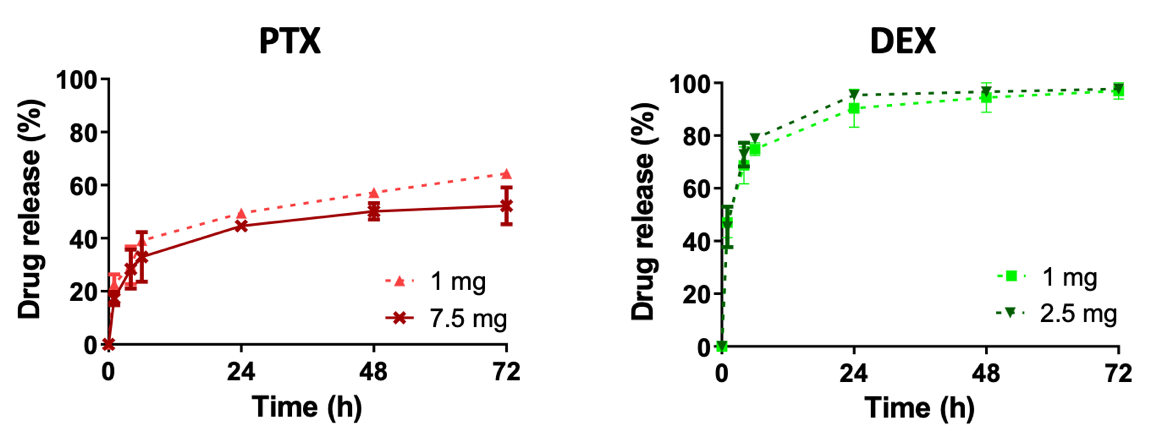
**

**Figure S6. Drug release profile of paclitaxel and dexamethasone single-loaded medium-size micelles using two different feed amounts.** Drug release was carried out under sink conditions using 45 mg/mL BSA solution in PBS (pH 7.4) as the medium. To prepare 1 mL of micellar dispersion, 30 mg of polymer were used. For paclitaxel (PTX), feed amounts of 7.5 and 1 mg were used, and for dexamethasone (DEX), feed amounts were 2.5 and 1 mg.


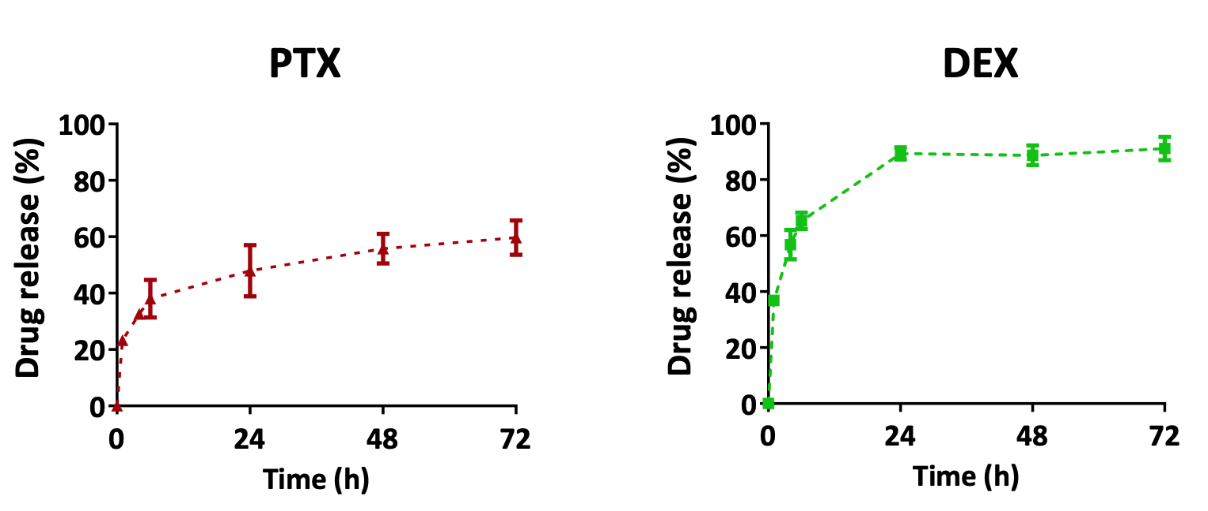


**Figure S7. Drug release profile from paclitaxel and dexamethasone single-loaded small-sized micelles.** Drug release was carried out under sink conditions using 45 mg/mL BSA solution in PBS (pH 7.4) as the medium. To prepare 1 mL of micellar dispersion, 30 mg of polymer were used. For paclitaxel (PTX), a feed amount of 7.5 was used, and for dexamethasone (DEX), the feed amount was 1 mg.

**
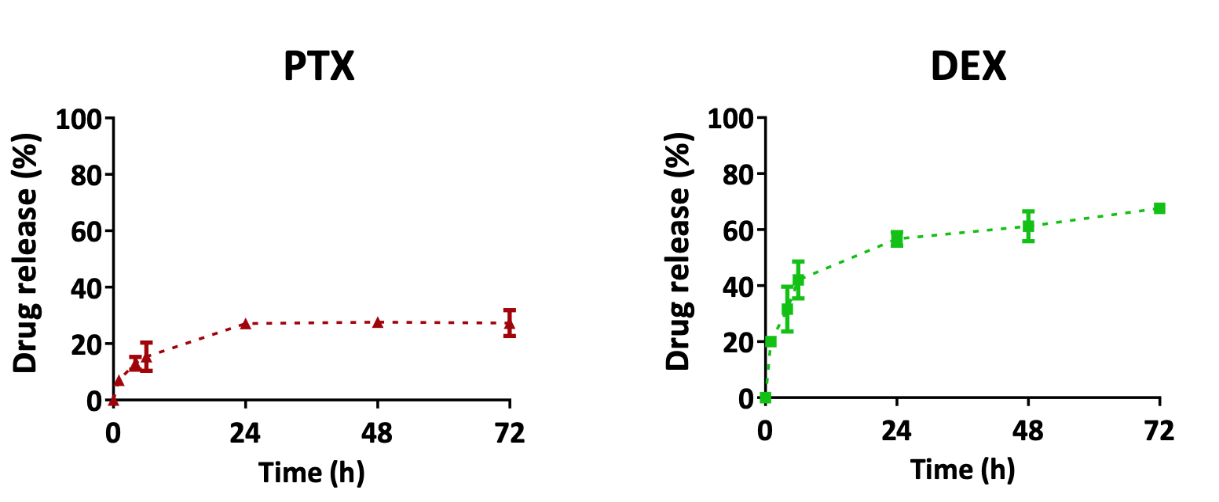
**

**Figure S8. Drug release profile from paclitaxel and dexamethasone single-loaded large-sized micelles.** Drug release was carried out under sink conditions using 45 mg/mL BSA solution in PBS (pH 7.4) as the medium. To prepare 1 mL of micellar dispersion, 30 mg of polymer were used. For paclitaxel (PTX), a feed amount of 7.5 was used, and for dexamethasone (DEX), the feed amount was 1 mg.

**Figure S9. Stability of and drug retention in co-loaded micelles under two pH conditions.** Stability of the micelles (size, PDI) and drug retention were evaluated over a period of 7 days. The experiments were performed in PBS-containing media at pH values 7.4 and 6. To prepare 1 mL of micellar dispersion, 30 mg of polymer were used. For taxanes (DTX, PTX, CTX), a feed amount of 7.5 mg was used. For corticosteroids (PRD, DEX, CIC), a feed amount of 1 mg was used.

**
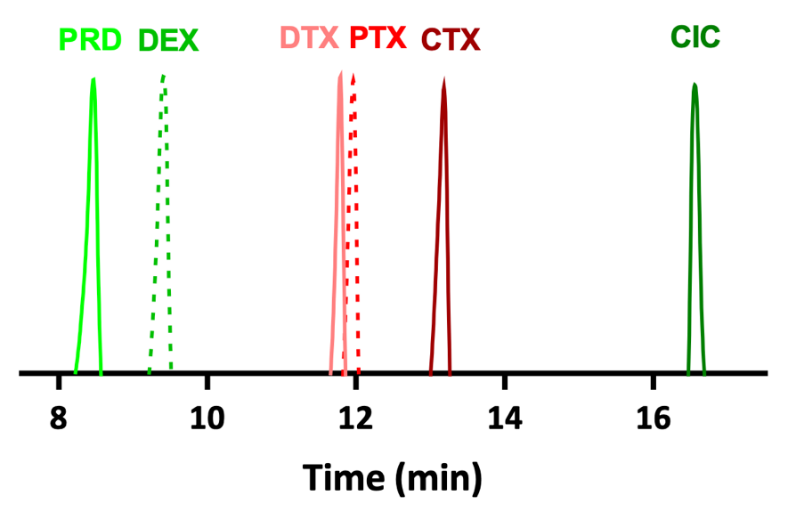
**

**Figure S10. HPLC chromatograms of the different taxanes and corticosteroids, using a non-polar C18 column and a 0 to 100 % gradient of ACN in water.** Retention times are as follows: prednisolone (PRD), 8.5 min; dexamethasone (DEX), 9.4 min; docetaxel (DTX), 11.8 min; paclitaxel (PTX), 12.0 min; cabazitaxel (CTX), 13.1 min; and ciclesonide (CIC), 16.4 min.

**Figure S11. Associations between drug properties and its retention in the micelles after 6 h.** Micellar drug retention after 6 h as a function of log P (**A**), water solubility (**B**), molecular weight (MW) (**C**), number of aromatic rings (**D**), and number of π electrons (**E**). Prednisolone (PRD), dexamethasone (DEX), ciclesonide (CIC), docetaxel (DTX), paclitaxel (PTX) and cabazitaxel (CTX) were the evaluated drugs.
